# Possible Mechanisms Linking Obesity, Steroidogenesis, and Skeletal Muscle Dysfunction

**DOI:** 10.3390/life13061415

**Published:** 2023-06-19

**Authors:** Anna F. Sheptulina, Karina Yu Antyukh, Anton R. Kiselev, Natalia P. Mitkovskaya, Oxana M. Drapkina

**Affiliations:** 1 Department of Fundamental and Applied Aspects of Obesity, National Medical Research Center for Therapy and Preventive Medicine, 101990 Moscow, Russia; sheptulina.anna@gmail.com (A.F.S.);; 2 Department of Therapy and Preventive Medicine, A.I. Evdokimov Moscow State University of Medicine and Dentistry, 127473 Moscow, Russia; 3 Republican Scientific and Practical Center of Cardiology, 220036 Minsk, Belarus; 4 National Medical Research Center for Therapy and Preventive Medicine, 101990 Moscow, Russia; 5 Department of Cardiology and Internal Diseases, Belarusian State Medical University, 220116 Minsk, Belarus

**Keywords:** obesity, steroidogenesis, sarcopenia, skeletal muscle dysfunction, insulin resistance, systemic inflammation

## Abstract

Increasing evidence suggests that skeletal muscles may play a role in the pathogenesis of obesity and associated conditions due to their impact on insulin resistance and systemic inflammation. Skeletal muscles, as well as adipose tissue, are largely recognized as endocrine organs, producing biologically active substances, such as myokines and adipokines. They may have either beneficial or harmful effects on the organism and its functions, acting through the endocrine, paracrine, and autocrine pathways. Moreover, the collocation of adipose tissue and skeletal muscles, i.e., the amount of intramuscular, intermuscular, and visceral adipose depots, may be of major importance for metabolic health. Traditionally, the generalized and progressive loss of skeletal muscle mass and strength or physical function, named sarcopenia, has been thought to be associated with age. That is why most recently published papers are focused on the investigation of the effect of obesity on skeletal muscle function in older adults. However, accumulated data indicate that sarcopenia may arise in individuals with obesity at any age, so it seems important to clarify the possible mechanisms linking obesity and skeletal muscle dysfunction regardless of age. Since steroids, namely, glucocorticoids (GCs) and sex steroids, have a major impact on the amount and function of both adipose tissue and skeletal muscles, and are involved in the pathogenesis of obesity, in this review, we will also discuss the role of steroids in the interaction of these two metabolically active tissues in the course of obesity.

## 1. Introduction

The global prevalence of obesity has now reached epidemic proportions. This condition currently affects >2 billion people worldwide, and more than 1.9 billion adults globally are overweight [1]. According to the experts’ estimates, if the current incidence trend continues, by 2030, 60% of the world’s population (that is, 3.3 billion people) may be overweight or obese [2].

Obesity is a multifactorial condition, resulting mainly from the imbalance between energy intake and expenditure and leading to the excess accumulation of fat in the body. This, in turn, causes various metabolic and neurohumoral disturbances maintaining the vicious circle of excess fat accumulation and increasing the likelihood of a variety of conditions accompanied by high incidence of morbidity and mortality. They include, in particular, cardiovascular diseases, type 2 diabetes mellitus (T2DM) [3,4], chronic kidney disease [5], non-alcoholic fatty liver disease [6], diseases of the musculoskeletal system [7], depression [8], and different types of cancer [9]. The pathophysiology of obesity is considered to be complex and to include the interaction between genetic, environmental, neurohormonal, microbial, and psychosocial factors [2,4,10]. For a long period of time, it was believed that all of the negative health effects associated with obesity are due to the excess accumulation of adipose tissue in the body. However, increasing evidence suggests that a decrease in skeletal muscle mass and quality, as well as skeletal muscle function, may also be relevant. Muscles play an important role in energy homeostasis, and they may be involved in the pathogenesis of obesity due to their impact on insulin resistance, energy expenditure, and systemic inflammation [10].

Generally, the loss of muscle mass and function, i.e., sarcopenia, was considered a feature of aging. However, according to the ESPEN and EASO Consensus Statement on the Definition and Diagnostic Criteria for Sarcopenic Obesity [11], sarcopenia may arise in individuals with obesity at any age. Indeed, T.L. Silva et al. [12] showed that the prevalence of sarcopenia among young and middle-aged adults (*n* = 108, mean age: 43 ± 11.7 years) of both sexes ranged between 11.1% and 13.9% depending on the criteria being used for the diagnosis of sarcopenia (low muscle mass or low muscle strength, correspondingly). Additionally, in the study conducted by E. Poggiogalle et al. [13], the prevalence of sarcopenia in a cohort of 727 participants with obesity (141 males, mean age: 45.6 ± 13.5 years; 586 females, mean age 45.8 ± 13.6 years) was 34.8% in males and 50.1% in females. This condition, characterized by the simultaneous presence of sarcopenia and obesity, was named sarcopenic obesity, and is believed to be associated with worse health outcomes than obesity and sarcopenia on their own, including the higher incidence of osteoporosis [11,14].

It is well known that serum glucocorticoid (GC) levels are elevated in individuals with obesity, and this contributes to the development of insulin resistance, hyperglycemia, and dyslipidemia [15]. The accompanied increase in circulating free fatty acids may result in the lipid infiltration of the liver, pancreas, and skeletal muscles, leading to the deterioration of their function. Obesity is also associated with disturbances in sex steroid synthesis and signaling, negatively affecting fertility [16,17]. On the other hand, owing to the well-described effects of androgens and estrogens on muscle mass, structure, and function, the impaired sex steroid balance in obesity may result in the deterioration of muscle health [18]. The fatty infiltration of skeletal muscles is defined as myosteatosis, and is characterized by the decrease in muscle strength, regeneration capacity, and the profile of biologically active substances produced by myocytes, i.e., myokines. It was shown that myokines acting together with other organokines, such as adipokines and hepatokines, may take part in the pathogenesis of obesity and associated conditions [19,20]. Currently, they are extensively studied as potential prognostic markers and therapeutic targets [20,21]. We provide a comprehensive review on the structural and functional changes of skeletal muscle in the course of obesity, as well as the impact of steroids on this process. To the best of our knowledge, this is the first multi-faceted review focused on the interactions among adipose tissue, skeletal muscle, and steroids in patients with obesity.

## 2. Skeletal Muscles in Obesity

**Figure 1:**
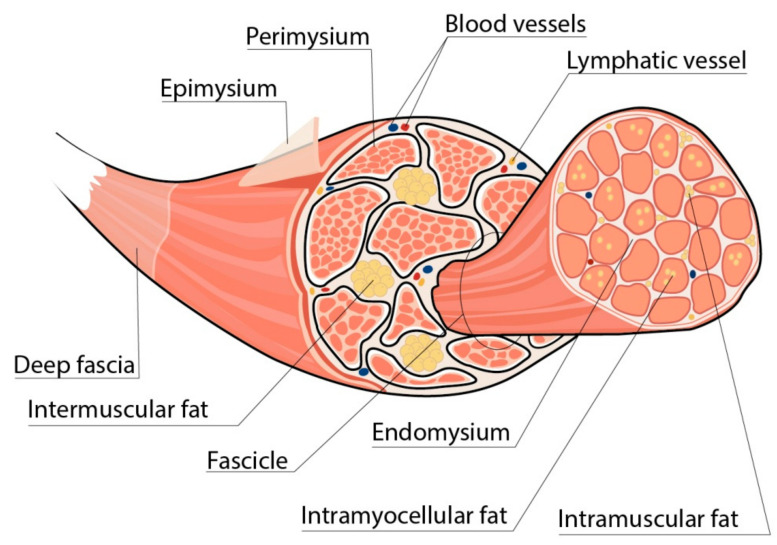
Localization of fat depots in skeletal muscles. Intermuscular fat depots represent adipocytes localized between muscle groups, whereas intramuscular fat depots are adipocytes located between the muscle fibers. Usually, these two depots are combined by the term “intermuscular fat”. Intramyocellular lipids denote lipid droplets stored within the myocytes.

There are three main adipose tissue depots in the body: subcutaneous, visceral, and ectopic. Ectopic corresponds to the accumulation of adipose tissue in internal organs or other compartments of the body, including skeletal muscles, which are originally unappropriated for the storage of fat [14]. Depending on the localization, fat depots in skeletal muscles may be classified as intermuscular (adipocytes localized between muscle groups), intramuscular (adipocytes located between the muscle fibers), and intramyocellular (IMCL, lipids stored within the myocytes) (Figure 1) [22]. Usually, adipocytes localized between the muscle fibers (intramuscular fat) and muscle groups (literally intermuscular fat) are combined by the term “intermuscular” (INTM) fat as both of them represent the fat depots situated underneath the deep fascia of the muscle, are formed by adipocytes of the same origin, and have similar significance for the muscle metabolic state [23]. In this review, we will also use the term “intermuscular fat” to denote both depots.

### 2.1. Metabolic Properties of INTM Fat

Currently, INTM fat is most often considered to be related to metabolic disturbances. Indeed, several studies have established that the increase in the amount of INTM fat is significantly associated with a decrease in muscle mass and strength, as well as insulin sensitivity [24,25]. Several studies indicate that INTM fat is functionally quite similar to visceral adipose tissue (VAT), particularly with regard to its ability to induce and maintain inflammation and influence insulin sensitivity [24,26]. For instance, both INTM fat and VAT are characterized by the increased expression of interleukin (IL)-6, tumor necrosis factor (TNF), and plasminogen activator inhibitor 1 (PAI1) [27]. At the same time, the results of direct comparisons of the qualitive and quantitative composition of inflammatory proteins secreted by INTM fat, VAT, or subcutaneous adipose tissue (SAT) in individuals with obesity clearly indicate that INTM depots secrete substantially greater amounts of inflammatory cytokines (such as interferon-(IFN) γ, IL-2, IL-5, and IL-10) and chemokines compared to VAT or SAT [28]. Moreover, it was shown that the lipolytic capacity of INTM fat was significantly greater than the rate of lipolysis in subcutaneous adipose tissue and was similar to that in VAT [29,30]. Thus, INTM fat may increase the concentration of free fatty acids in the interstitial space, negatively influencing the local muscle microenvironment and promoting muscle insulin resistance. In an in vitro study, it was shown that proteins secreted by INTM fat were able to increase the concentration of 1,2-diacylglycerols within the myotubes, intramyocellular lipids associated with impaired insulin sensitivity [29]. Sachs et al. [26] demonstrated that the metabolic properties of INTM fat can change and become more unfavorable with the aggravation of metabolic health. Further, taking into account the role of insulin signaling in muscle protein synthesis, the impaired insulin sensitivity of skeletal muscles resulting from myosteatosis may directly contribute to the decrease in muscle strength and mass [31].

It is suggested that INTM fat may originate from several cell sources, in particular fibro-adipogenic progenitors, muscle satellite stem cells, adipose-derived stem cells, and bone-marrow-derived mesenchymal stem cells.

#### 2.1.1. Fibro-Adipogenic Progenitors

It is believed that adipocytes constituting the INTM depots originate mainly from fibro-adipogenic progenitors (FAPs) [31]. These cells received their name because of their ability to differentiate either into fibroblasts or adipocytes. In in vitro studies, it was shown that the fibrogenic differentiation of FAPs can be induced by transforming growth factor-β (TGF-β), whereas their differentiation into adipocytes can be stimulated by insulin, 3-isobutyl-1-methylxanthine, and dexamethasone [32,33,34]. Except for being involved in muscle tissue regeneration under physiological conditions [35], these cells take part in the processes of muscle atrophy during disease and aging [36,37]. Moreover, animal studies showed that FAPs are essential for the maintenance of the amount and function of muscle stem cells, which are responsible for muscle regeneration and growth [38,39].

It is suggested that FAP differentiation into fibroblasts or adipocytes in the settings of insulin resistance and impaired metabolism can contribute to the muscle remodeling, degeneration, and fibrosis taking place in obesity [40]. For instance, it was shown that thrombospondin 1 (THBS1), an adipokine secreted by adipocytes of white adipose tissue, is capable of promoting the proliferation of FAPs in obese mice [41]. In addition, chronic inflammation, being an important pathogenic mechanism in the development of obesity and associated conditions, may sustain the activated state of FAP, thus contributing to the development and progression of the so-called fibrofatty degeneration of skeletal muscles in obesity. It is worth noting that this condition is accompanied not only by the decrease in muscle function, but also by the increase in the insulin resistance of the muscle tissue. Such effects may be due to the cytokines expressed either by M1 (IL-1β polarized) or M2 (IL-4 polarized) macrophages infiltrating muscle tissue, and their influence on the processes of FAP differentiation and adipogenesis [42]. Finally, it was shown that adipocytes derived from FAPs were characterized by the impaired phosphorylation of insulin receptors, which contributed to the increase in peripheral insulin resistance [43].

#### 2.1.2. Other Cell Types as Possible Source of INTM Fat

B.H. Goodpaster et al. [25] suggested that, along with FAPs, several other cell types may participate in the generation of INTM fat depots, including muscle satellite stem cells (MSCs) and adipose-derived stem cells (ASCs), which migrate into the muscles from other adipose tissue depots. Although MSCs represent a major source of skeletal muscle cells and are responsible for muscle tissue regeneration and growth in the postnatal period, in vitro studies have shown that MSCs do differentiate into adipocytes in the presence of adipogenic factors [44,45]. However, the exact factors capable of stimulating the MSC differentiation into adipocytes in vivo remain unknown. For instance, L. Guo et al. [46] showed that intramuscular preadipocytes might hamper the differentiation of MSCs via C-Jun N-terminal kinases (JNK)/mitogen-activated protein kinase (MAPK) pathway and simultaneously facilitate their lipid deposition through peroxisome proliferator–activated receptor (PPAR) signaling in chickens.

Finally, ASCs and bone-marrow-derived mesenchymal stem cells constitute the third and even fourth possible sources of INTM fat depots, though the exact mechanisms, as well as factors contributing to their migration into the muscle tissue bed, require further investigation [47,48].

### 2.2. Intramyocellular Fat Depots

Intramyocellular (IMCL) fat depots refer to the accumulation of fat within the myofibers themselves. Inside the muscle fibers, lipids are stored mainly in the form of triacylglycerols (TAG), being localized within the lipid droplets. Other components of such lipid droplets include diacylglycerols (DAG), sphingolipids, and phospholipids. These depots are thought to be important for muscle contraction during physical exercises and to be associated with the impaired insulin sensitivity in patients with obesity and/or T2DM, as well as in elderly and sedentary individuals. Indeed, in their study, J.J. Dubé et al. [49] showed that the completion of a 16-week moderate exercise training program was accompanied by the increase in IMCL fat content by 21% in 25 older obese individuals. Additionally, the authors noticed that physical activity contributed to the increase in the content of triacylglycerols in skeletal muscles and to the decrease in the content of both diacylglycerols and ceramides within the IMCL depots. This, in turn, resulted in the improvement of insulin sensitivity. It is worth noting that the above-mentioned effect was independent of weight reduction, thus suggesting that physical activity itself may have a beneficial effect on the metabolic health of obese adults.

**Figure 2:**
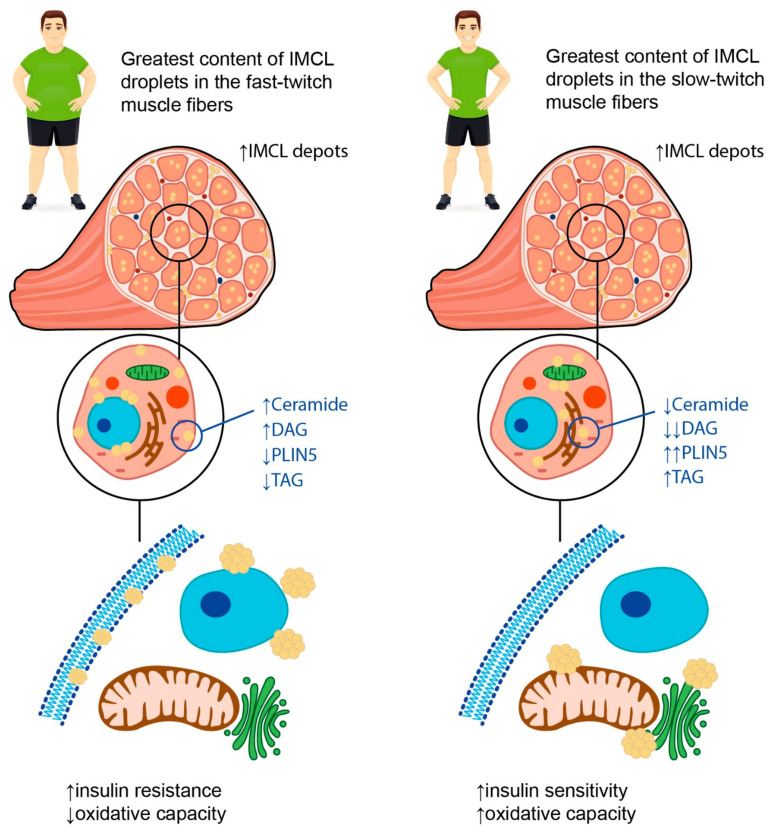
Possible explanations for “athlete’s paradox”. The term “athlete’s paradox” describes a condition characterized by high amounts of IMCL fat in the muscles observed both in patients with obesity and/or T2DM, and in endurance-trained athletes. In obese individuals, the IMCL depots are located mainly in fast-twitch muscle fibers and contain high amounts of ceramides and diacylglycerols (DAGs), as well as low concentrations of perilipin 5 (PLIN5) and triacyclglycerols (TAGs). In contrast, in endurance-trained athletes, IMCL droplets are situated primarily in slow-twitch muscle fibers and carry high amounts of TAGs and PLIN5 along with low concentrations of lipid metabolites, such as DAGs and ceramides. The intracellular localization of IMCL depots also differs between endurance-trained athletes and patients with obesity. In particular, in athletes, lipid droplets are located predominantly in close relation to mitochondria and the endoplasmic reticulum, while in obese individuals, they may be found in the sarcolemma, subsarcolemmal, or perinuclear regions. Overall, these differences determine a decrease in muscle oxidative capacity and insulin sensitivity in obese patients in contrast to the athletes, despite similar numbers of IMCL depots within the muscle fibers. Up-oriented arrows (↑) indicate up-regulation. Down-oriented arrows (↓) indicate downregulation. The number of arrows corresponds to the magnitude of the differences.

These described findings are in line with the so called “athlete’s paradox”; that is, the higher amounts of IMCL fat in the muscles are observed not only in patients with obesity and/or T2DM, but also in endurance-trained athletes (Figure 2) [49,50,51]. There are several possible explanations for this phenomenon. First, the increase in the IMCL fat amount in trained athletes is necessary to provide enough substrates for energy metabolism during regular physical exercise, whereas in obese patients and patients with T2DM, the IMCL depots serve as storage for excess fat [52]. Second, the content of IMCL depots does differ between the endurance-trained athletes and obese adults. In particular, the latter have greater amounts of DAGs and ceramides within the IMCL depots. These substances are regarded as lipid metabolites, and the increase in their content within the myofibers is suggested to be associated with impaired insulin sensitivity, possibly due to the decrease in muscle oxidative capacity [49]. Accordingly, B.C. Bergman et al. [51] demonstrated that physical exercise promoted the decrease in di-saturated DAG concentration both within the membrane and cytosol, and this resulted in the insulin sensitization of skeletal muscles. Moreover, A Gemmink et al. [53] showed that trained athletes had higher concentrations of perilipin 5 (PLIN5), a lipid droplet coating protein, compared to patients with T2DM. Although the content of PLIN5 was not significantly associated with the size and number of lipid droplets, it was positively correlated with the muscle oxidative capacity.

Third, the rate of TAG synthesis within the myofibers differs between the endurance-trained athletes, insulin resistant obese patients, and lean sedentary individuals, being the highest in the former. These data suggest that high rates of IMCL fat synthesis, which influences intramuscular lipid structuring and localization, can help to prevent the formation of insulin resistance [50,51]. In relation to lipid droplet partitioning, it was established that the accumulation of triglycerides in the sarcolemma and nucleus was negatively corelated with insulin sensitivity, whereas the localization of IMCL fat depots in close relation to mitochondria and the endoplasmic reticulum was not associated with the development of insulin resistance and appeared to facilitate the oxidation of TAGs [51,54]. Similar results were obtained in the study by M.C. Devries et al. [55] that included 11 obese and 12 lean sedentary women who underwent the 12-week endurance training program. Though the endurance training did not result in weight loss, it led to the increase in muscle oxidative capacity. Additionally, after the completion of the endurance training program, the localization of the IMCL fat depots changed in both groups: the content of IMCL lipid droplets in the subsarcolemmal region reduced, whereas the content of IMCL fat depots in the intermyofibrillar compartment increased. Finally, the saturated vs. unsaturated fatty acid ratio within the IMCL fat depots may also be relevant. Indeed, D. Kahn et al. [54] showed that patients with T2DM had the highest content of saturated fatty acids within the myofibers, and this appeared to be significantly associated with insulin resistance.

Interestingly, the degree of lipid accumulation within IMCL depots in obesity may depend on the type of muscle fibers. In their study, N. Umek et al. [56] demonstrated that the fast-twitch muscles of obese mice (i.e., gastrocnemius and intermediate plantaris muscles) were characterized by the greatest content of IMCL lipid droplets, while in slow-twitch muscles (soleus muscle), there was no significant lipid accumulation. Furthermore, the authors described an increase in the expression of fast-type myosin heavy chain in the slow-twitch soleus muscles of the obese mice.

In summary, one can conclude that not only the amount, but also the composition and localization of IMCL fat depots within the myofibers may be crucial for the development of insulin resistance and the deterioration of metabolic health in obese patients. Additionally, the capacity of skeletal muscles for lipid oxidation may be the factor determining the association between IMCL fat content and insulin resistance and may depend on muscle composition as well as metabolic flexibility.

### 2.3. Skeletal Muscle Composition and Metabolic Flexibility in Obesity

Skeletal muscle metabolism is crucial for total daily energy expenditure; along with the brain, liver, heart, and gastrointestinal tract, skeletal muscles are regarded as major contributors to overall metabolic rate [57]. This is due to their participation in resting energy expenditure and ability to increase energy expenditure with physical exercise [58]. Depending on metabolic activity, fatigability, mitochondrial content, and movement rates, muscle fibers may be classified into two different types: type I (slow-twitch or oxidative) and type II (fast-twitch or glycolytic) muscle fibers [59,60]. Type II fibers can be further classified as type IIa fibers, also called fast-oxidative glycolytic fibers or intermediate fibers, and IIx fibers, also termed fast glycolytic fibers. The latter contain high amounts of glycogen and use primarily anaerobic metabolism to generate adenosine triphosphate (ATP), so they are characterized by relatively small numbers of mitochondria [59,61]. It is believed that the distribution of muscle fiber types within muscle groups varies substantially among individuals and may be influenced by both environmental (nutrition, physical exercises, etc.) and biological factors (age, gender, genetics, etc.), thus determining the individual differences in daily energy expenditure, as well as the individual risk for sustaining a positive energy balance, and, as a consequence, weight gain [58,61]. Indeed, it was established that patients with obesity have lower proportions of type I muscle fibers characterized by an increased number of mitochondria and, at the same time, greater proportions of type IIx fibers compared with lean individuals [61,62,63]. These data are confirmed by the results of D.A. Reiter et al., suggesting that obesity is associated with reduced muscle energetic efficiency with a predominance of glycolysis over oxidation processes [64].

In addition to disturbed proportions of different muscle fibers, mitochondria content and muscle tissue metabolism are also altered in obesity. To define the ability of muscle fibers to use either glucose or fatty acids as a fuel depending on the availability of the substrates, physical activity, and conditional changes in metabolic or energy demand, the term “muscle metabolic flexibility” was proposed [65]. This concept, named the Randle Cycle, was first suggested by P.J. Randle et al. in 1963 [66] to describe the fuel selection in the transition from fasting to fed states and to characterize its role in insulin sensitivity and metabolic health. Nowadays, the term “metabolic flexibility” is used with respect to physiological adaptability and encompasses several other tissues and metabolic circumstances.

Increasing evidence suggests that an inability to adapt to metabolic stimuli, such as insulin signaling or fatty acid exposure, may lead to the decrease in muscle oxidative capacity characteristic of obesity [67,68,69]. In their study including 28 juvenile Iberian pigs fed either a control or a high-fructose, high-fat (HFF) diet for 10 weeks, H.C. Spooner et al. [70] demonstrated that the HFF diet resulted in decreased IMCL fat content and in the formation of less oxidative skeletal muscle phenotype, reflecting the disturbed ability of skeletal muscles to use lipids as a fuel. These changes were similar to the effects of detraining or muscle atrophy, indicating the reduced capacity of muscles to perform endurance-type exercises [71]. These effects may be followed by a long-term decrease in muscle mass and strength. Indeed, G.V. Hernandez et al. [72] showed that there was a reduction in plasma creatinine levels in juvenile Iberian pigs fed an HFF diet during 10 weeks, suggesting a tendency towards deterioration in muscle growth in this animal model.

**Figure 3:**
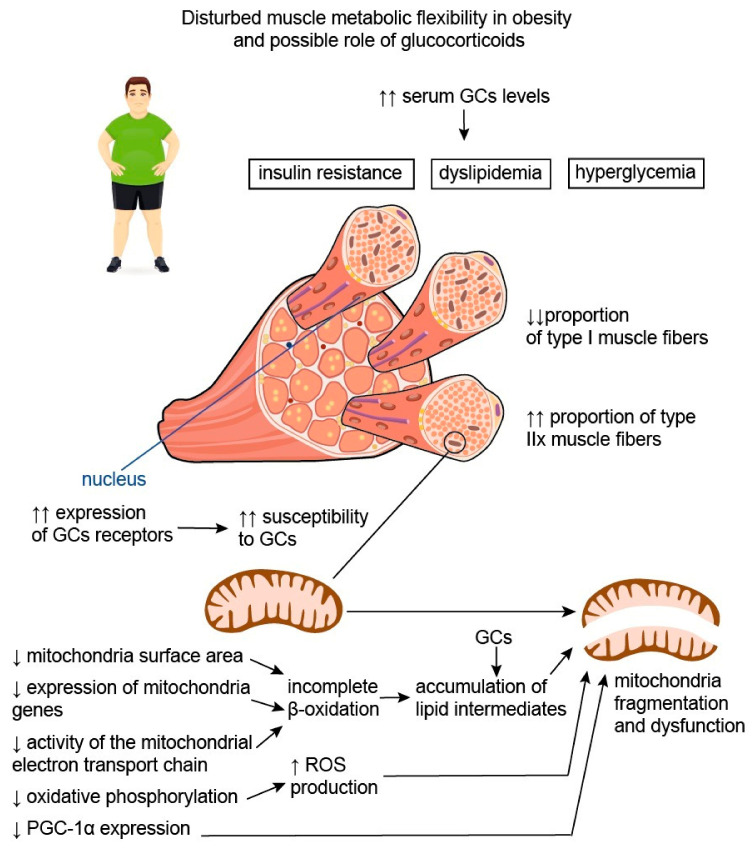
Disturbed muscle metabolic flexibility in obesity and possible role of glucocorticoids. Disturbed muscle oxidative capacity is believed to be associated with both the change in the proportions of muscle fiber types and mitochondrial dysfunction. Patients with obesity have lower proportions of type I muscle fibers characterized by an increased number of mitochondria and, at the same time, greater proportions of type IIx fibers compared with lean individuals. Thus, obesity is associated with reduced muscle energetic efficiency with a predominance of glycolysis over oxidation processes. In addition, obesity is characterized by the decrease in mitochondrial surface area, substantially lower expression of mitochondria genes, and reduced activity of the mitochondrial electron transport chain. This causes incomplete β-oxidation and, consequently, the accumulation of lipid intermediates, capable of affecting mitochondrial morphology and function. Disturbed oxidative phosphorylation leads to the increased production of reactive oxygen species (ROS), which facilitate mitochondrial fragmentation. In these settings, the expression of peroxisome proliferator-activated receptor–gamma coactivator-1alpha (PGC-1α), which is initially lower in type IIx fibers compared to type I fibers, is being downregulated, further aggravating mitochondrial damage and dysfunction. It is well known that serum glucocorticoid (GC) levels are elevated in individuals with obesity, and this contributes to the development of insulin resistance, hyperglycemia, and dyslipidemia. Moreover, GCs appear to be involved in the disturbance of muscle metabolic flexibility in obesity. It is established that type IIx muscle fibers, being the predominant muscle fiber type in patients with obesity, are characterized by greater susceptibility to GCs, possibly due to the greater expression of GC receptors. Therefore, the negative effects of GCs on skeletal muscle may be more pronounced in patients with obesity. Additionally, GCs are known to increase the accumulation of lipid intermediates in skeletal muscle, thus facilitating mitochondria dysfunction and impairment of muscle energy metabolism. Up-oriented arrows (↑) indicate up-regulation. Down-oriented arrows (↓) indicate downregulation. The number of arrows corresponds to the magnitude of the differences.

Disturbed muscle oxidative capacity is believed to be associated with both changes in the proportions of muscle fiber types and mitochondrial dysfunction (Figure 3). It was shown that obesity is accompanied by a decrease in mitochondrial surface area by, on average, 20–60%, as well as by the substantially lower expression of mitochondrial genes and metabolites [67,68,73]. Accumulating data suggest that obesity has an unfavorable impact on the activity of the mitochondrial electron transport chain and oxidative phosphorylation, as well as stimulating the production of reactive oxygen species (ROS), facilitating mitochondrial fragmentation and mutations in mitochondrial DNA [74]. This may be due to mitochondria lipid overload, which causes incomplete β-oxidation and, consequently, the accumulation of lipid intermediates, such as ceramides. It was shown that ceramides could affect membrane potential, electron transport, and mitochondrial morphology, resulting in mitochondrial dysfunction [74]. In these settings, the expression of peroxisome proliferator-activated receptor–gamma coactivator-1alpha (PGC-1α), known to be involved in mitochondrial biogenesis, and to exert antioxidant action is being downregulated in order to prevent further mitochondrial function deterioration due to the accumulation of damaged mitochondrial DNA [75]. PGC-1α is also known to regulate lipid oxidation, energy homeostasis, and insulin sensitivity. In particular, it was demonstrated that the inactivation of PGC-1α expression in skeletal muscles in mice led to a shift from oxidative type I and IIa muscle fibers toward type IIx and IIb glycolytic muscle fibers [76]. On the contrary, elevated levels of PGC-1α in the skeletal muscles of transgenic mice were shown to protect against age-related obesity and T2DM [77]. The results of these studies reflect the important role of PGC-1α in promoting effective muscle energy metabolism and regulating energy balance in the body. 

Interestingly, weight loss does not necessary lead to an increase in mitochondrial number and the recovery of mitochondrial function. In their study, E.V. Menshikova et al. [78] assessed the impact of weight loss through calorie restriction (*n* = 7) or moderate-intensity exercise (*n* = 10) on skeletal muscle mitochondrial content, mitochondrial enzyme activities, and insulin resistance in 17 overweight or obese individuals aged 60–75 years. Interestingly, although both interventions resulted in weight loss and improved insulin sensitivity, only physical activity was associated with an increase in mitochondria content and the activity of enzymes involved in mitochondrial electron transport chain and fatty acid oxidation within the skeletal muscle.

### 2.4. Myokines in Obesity

Recently it was established that skeletal muscles produce some biologically active substances called myokines, which facilitate the crosstalk between muscles and some other organs and tissues, such as adipose tissue, brain, liver, bone, and kidney. The production and secretion of nearly all such molecules, namely, irisin/fibronectin type III domain-containing protein 5 (FNDC5), IL-15, IL-6, brain-derived neurotrophic factor (BDNF), and myonectin, are upregulated during muscle contraction and physical exercise [75]. Here, we will discuss the role of the two most studied PGC-1α-dependent myokines, irisin and myostatin, in the deterioration of muscle structure and function in obesity.

#### 2.4.1. Irisin

A recent meta-analysis concluded that circulating irisin levels were higher in obese individuals compared to healthy controls, though they appeared to be affected by ethnicity and age [79]. Irisin is produced by the proteolytical cleavage of FNDC5 during muscle contraction and physical exercise. It is a PGC-1α-dependent myokine, able to increase the browning of white adipose tissue by increasing the expression of uncoupling protein 1 (UCP-1). Adipose tissue and liver are also able to secret this molecule in small amounts [80]. The elevation in circulating irisin levels in patients with obesity without T2DM may be explained by the existence of obesity-induced metabolic dysfunction, particularly insulin resistance. In this case elevated irisin levels may represent an attempt to maximize glucose uptake by skeletal muscle and prevent hyperglycemia [81,82]. Interestingly, it was shown that after the onset of T2DM, the expression of FNDC5 in the muscle of treatment-naïve patients in vivo was reduced by ~15%. However, in in vitro experiments, myotubes isolated from these patients were capable of expressing FNDC5 in greater amounts than myotubes taken from lean individuals [81]. In contrast, it was shown that the irisin secretion from adipocytes in patients with obesity is lower than in lean controls [81,83]. However, as the body fat mass is substantially increased in patients with obesity, the overall levels of irisin secreted by adipose tissue in patients with obesity may be quite similar or even superior to those in lean individuals.

It is worth noting that plasma irisin levels may be significantly decreased following weight loss due to bariatric surgery. This effect may be explained by the decrease in fat-free mass during weight loss and, as a consequence, lower FNDC5 mRNA expression in skeletal muscle. In support of this suggestion, the irisin concentration returned to baseline levels in patients who regained the original weight [84,85]. However, the contribution of adipose tissue to the increase in total irisin levels upon weight regain cannot be excluded. These data also confirm the notion that an increase in irisin levels in patients with obesity may mirror metabolic disturbances characteristic of these patients, and may be directed at the compensation of these abnormalities [20].

According to J. Jia et al. [79], irisin levels were higher in young participants compared to older ones, possibly due to the age-related decline in muscle function. These authors further found out that irisin levels were higher in obese individuals compared to controls when they were from Africa, while no significant differences were described in European, Asian, or American populations. These inconsistencies may result from genetic factors capable of influencing the levels of irisin, as well as from the differences in body mass index (BMI) criteria used to diagnose overweight and obesity in the Asian population.

Moreover, it was shown that irisin expression may be associated with some anti-inflammatory markers [86]. In particular, irisin was able to suppress the expression of pro-inflammatory cytokines, nuclear factor-kappa B (NF-κB), TNF-α, and IL-6. In addition, irisin reduced the monocyte chemoattractant protein 1 (MCP-1) expression in cultured adipocytes with the subsequent attenuation of macrophage migration in the presence of irisin. Irisin was also able to induce the transition from the M1 (pro-inflammatory) macrophage phenotype to the M2 (anti-inflammatory) phenotype [86,87].

Therefore, taking into account the above-mentioned information, irisin seems to play a protective role in the development of metabolic dysfunction in obesity directed at the attenuation of insulin resistance and inflammation, as well as at the improvement of energy metabolism. However, its concentration may be decreased in case of sarcopenic obesity.

#### 2.4.2. Myostatin

Myostatin, also known as growth and differentiation factor 8 (GDF8), is a member of the TGF-β superfamily. It is expressed predominantly in skeletal muscle, and its secretion and production are inhibited during muscle contraction and exercise [88]. Except for being involved in muscle atrophy [89], myostatin is regarded as a potential modulator of metabolic homeostasis acting through the regulation of adipose tissue function [90,91]. With regard to its effects, myostatin may be considered an irisin antagonist. For instance, it was demonstrated that the inhibition of myostatin was able to decelerate the development of insulin resistance and obesity in mice fed a high-fat diet, possibly due to the intensification of lipolysis and mitochondrial lipid oxidation in liver and adipose tissue. Moreover, it was shown that the inactivation of myostatin resulted in the formation of brown adipose tissue in the white adipose tissue of myostatin knockout mice [92]. This mouse line was also characterized by the upregulation of the expression and phosphorylation of adenosine monophosphate (AMP)-activated protein kinase (AMPK) in muscle, leading to the activation of PGC-1α and FNDC5 [93]. A study by M. Amor et al. reported an increase in serum myostatin concentrations in patients with obesity compared to lean individuals with no differences in myostatin expression in adipose tissue [90]. The authors showed that circulating myostatin levels were positively correlated with insulin resistance, while muscle myostatin gene expression was strongly associated with the expression of metabolic genes, particularly the insulin receptor substrate-1 (IRS-1) gene and sterol regulatory element binding transcription factor 1 (SREBTF1) gene [90]. Interestingly, the inhibition of myostatin expression in mice was accompanied by an improvement in insulin sensitivity: myostatin knockout mice were even more insulin sensitive than wild-type mice, suggesting that myostatin’s effect on insulin signaling may be independent of muscle mass [94]. Accordingly, physical exercises, bariatric surgery [95], and a calorie-restricted Dietary Approaches to Stop Hypertension (DASH) diet [96], due to their well-known beneficial effects on insulin sensitivity, were demonstrated to suppress myostatin production in skeletal muscles.

Accumulating data suggest that myostatin is expressed in the human reproductive system and may have different functions. For instance, myostatin is involved in the regulation of steroidogenesis, cell proliferation, the formation of extracellular matrix, and gonadotrophin responsiveness, thus influencing the process of cell differentiation in emerging follicles [97]. In addition, it is suggested that myostatin may be involved in the pathogenesis of some ovarian diseases, particularly polycystic ovary syndrome and ovarian hyperstimulation syndrome [98,99].

In view of the important role of myostatin in muscle atrophy, sarcopenia, and fat accumulation, this substance represents a promising direction for future research with respect to aging and obesity being associated with the growing incidence of chronic non-communicable diseases.

## 3. Steroids, Obesity, and Skeletal Muscles

### 3.1. Glucocorticoids

Glucocorticoids are considered to be major regulators of metabolism in the whole body. Skeletal muscle represents the key GC target tissue, where GCs primarily regulate protein and glucose metabolism [100]. The main metabolic consequence of GCs’ action in skeletal muscle is the increase in blood glucose concentration aiming to provide enough glucose to fuel the brain during stress. This may be achieved by the inhibition of glucose uptake by muscle cells, the decrease in protein synthesis and increase in proteolysis, and the suppression of glycogen synthesis in skeletal muscles [15,100].

A recent study A.Z. Shirif et al. [101] showed that the combination of a high-fructose diet and chronic stress resulted in the increased PPAR-α and PPAR-δ expression in the skeletal muscle of male rats after 9 weeks of exposure. This, in turn, led to the activation of β-oxidation and promoted the use of fatty acids instead of glucose as a fuel in skeletal muscles, concomitant accumulation of lipid intermediates, and induction of insulin resistance via the activation of inflammation [102]. Additionally, A.Z. Shirif et al. described a decrease in GC signaling in the skeletal muscles of fructose-fed stressed rats, which, in the author’s opinion, may predispose to the initiation and maintenance of lipid-induced inflammation in the muscle tissue, further affecting its metabolism [101].

Nowadays, GCs represent the first-line treatment for Duchenne muscular dystrophy (DMD), as they are able to slow the deterioration of muscle function and physical performance by reducing the inflammation [102]. Additionally, recent studies indicate that glucocorticoids may have either pro- or anti-adipogenic effects on FAPs, and this depends on the type of glucocorticoids used and on the culture conditions. For example, according to the results of in vitro studies, budesonide exerts anti-adipogenic properties when added to actively proliferating FAPs in the presence of adipogenic induction medium, in contrast with halcinonide and clobetasol, which do not influence adipogenesis [103].

It is considered that the effect of GCs on skeletal muscles may depend on the duration of administration and dosing regimen. For instance, it was established that short-term administration of GCs might be beneficial for physical performance and for muscle recovery from injury due to the anti-inflammatory properties of GCs [104,105]. Additionally, M. Quattrocelli et al. [106] showed that although both daily and pulsatile dosing regimens of GCs enhanced muscle repair after acute injury in dystrophic mice, only the pulsatile dosing regimen (once-weekly administration of GS over 4 weeks) was able to enhance muscle function and physical performance, while daily dosing of GCs resulted in muscle wasting. The authors documented that the negative effect of daily dosing was associated with the activation of atrophic pathways, particularly F-box protein 32 (Fbxo32) in skeletal muscles, while once-weekly dosing was accompanied by a decrease in Fbxo32 expression [106].

Chronic GC use is associated with negative consequences, namely, obesity and metabolic disturbances, as well as muscle atrophy [107,108]. The regulation of muscle atrophy induced by chronic GC exposure appears to be associated with the activity of enzyme 11-beta hydroxysteroid dehydrogenase 1 (11β-HSD1), which converts cortisone to cortisol. This enzyme is also involved in the development of GC-induced insulin resistance, a factor possibly contributing to muscle atrophy [109,110]. The effect of GCs on fast- and slow-twitch skeletal muscles is not similar: fast-twitch skeletal muscles are more susceptible to the negative impact of GCs (Figure 3). This phenomenon may have several explanations. First, slow-twitch skeletal muscles may have greater importance in life support, as they participate in the maintenance of posture and respiration. Second, the expression of the GC receptors differs between the fiber types, i.e., fast-twitch muscles are characterized by the abundant expression of GC receptors [111,112]. Third, the differences may be due to the expression of PGC-1α in the slow-twitch fibers, which is lacking in the fast-twitch fibers. This substance is believed to prevent the development of muscle atrophy induced by fasting [113] and to promote muscle tissue remodeling to a fiber-type composition characterized by greater oxidative (and, consequently, less glycolytic) capacity [114].

Taking into account the known sexual dimorphism of skeletal muscles, I. Salamone et al. [115] analyzed whether there are any differences in the muscle response to steroids between male and female mice. The authors showed that once-weekly GC exposure resulted in the increased expression of genes participating in the insulin growth factor 1 (IGF-1)/phosphoinositide 3-kinase (PI3K) pathway, which is known to be strongly implicated in muscle growth [116], as well as genes involved in calcium handling in male mice. In contrast, there was an upregulation of genes implicated in lipid metabolism in the skeletal muscles of female mice receiving GCs once-weekly during one month. Interestingly, the upregulated genes also have sexually dimorphic expression in skeletal muscles [117,118], and this may be due to the possible differences in fiber type, as female muscles appear to have a greater proportion of oxidative fibers compared to male muscles [119]. Moreover, weekly treated female mice exhibited a decrease in adipocyte size, whole-body percentage fat mass, and visceral fat pad size, while these changes were not described in treated male mice [115].

Other sex differences in muscle phenotypes between males and females are presented in Table 1.

### 3.2. Sex Steroid Hormones

**Figure 4:**
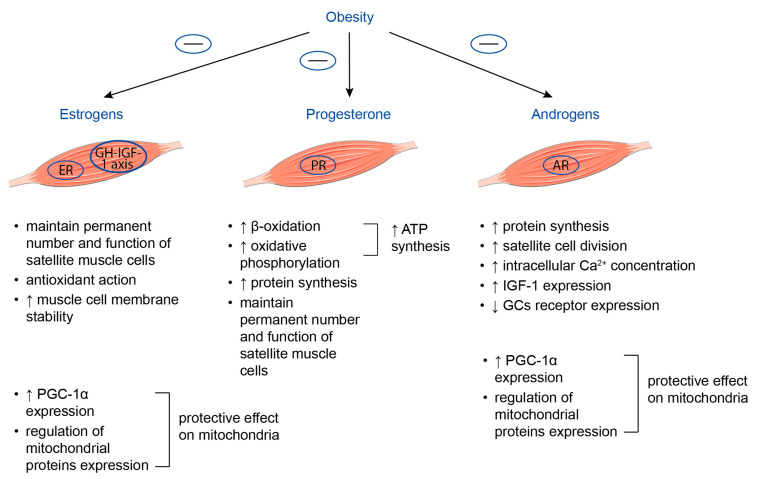
Key effects of estrogens, progesterone, and androgens on skeletal muscle. Obesity is known to be associated with reduced serum progesterone and estradiol concentrations in women, as well as with decreased production and secretion of androgens in men. Taking into account the beneficial effects of sex steroids on skeletal muscle, these changes in their serum concentrations may represent an additional mechanism contributing to the decline in muscle mass and function in obesity. AR = androgen receptor; ATP = adenosine triphosphate; ER = estrogen receptor; GCs = glucocorticoids; GH = growth hormone; IGF-1 = insulin growth factor 1; PGC-1α = peroxisome proliferator-activated receptor-gamma coactivator-1alpha; PR = progesterone receptor. Up-oriented arrows (↑) indicate up-regulation. Down-oriented arrows (↓) indicate downregulation.

Sex steroid hormones, namely, testosterone, estrogen, and progesterone, act as anabolic hormones and are involved in the maintenance of skeletal muscle mass and function (Figure 4) [128]. Estrogen and androgen receptors are expressed by muscle satellite cells, myoblasts, and myocytes, while progesterone receptors may be found at myocytes [129].

#### 3.2.1. Estrogens

It is considered that estrogen-mediated beneficial effects on skeletal muscle function, namely, the mitigation of muscle injury and enhancement of muscle repair after damage, may be associated with their ability to maintain a permanent number and function of satellite muscle cells, exert antioxidant action, particularly in the case of mitochondrial stress, and to increase muscle cell membrane stability. Additionally, estrogens are believed to participate in several signaling pathways, such as the modification of contractile proteins and apoptosis, involved in the preservation of muscle mass and strength [130]. Estrogens’ effects on skeletal muscle may be mediated by direct binding to their receptors situated at the level of muscle fibers, and indirectly through the regulation of the growth hormone–IGF-1 axis [131,132].

The decline in estradiol levels, characteristic of menopausal transition, is accompanied by the increased visceral adiposity as well as decreased muscle mass and strength and diminished bone mineral density [133]. Chronic low-intensive inflammation represents the pathophysiological mechanism common for all of the above-mentioned conditions. It was demonstrated that 17β-estradiol is capable of suppressing the production of several pro-inflammatory cytokines, namely, TNF-α, thus protecting skeletal muscle from wasting and facilitating recovery from injury [134,135]. It is worth noting that hormone replacement therapy containing estradiol was shown to be able to reverse both menopause-related obesity and loss of lean mass in healthy post-menopausal women, possibly due to the anabolic effect on skeletal muscle. Interestingly the overall body weight remained unchanged [136].

#### 3.2.2. Progesterone

Progesterone is another female sex hormone, whose concentration increases dramatically during the luteal phase of the menstrual cycle. Studies concerning the impact of endogenous progesterone on skeletal muscle structure and function are limited. Most of the data concerning the influence of progesterone on skeletal muscles come from studies in women taking hormone replacement therapy or oral contraceptives. Increasing evidence suggests that the binding of progesterone to its receptors found on the outer membrane of mitochondria results in the enhancement of beta-oxidation and oxidative phosphorylation, which leads to increased ATP synthesis, thus providing muscles with the energy necessary for the maintenance of their mass and function [137,138]. Moreover, progesterone may have a beneficial effect on muscle protein turnover in postmenopausal women. It was demonstrated that 100 mg/day progesterone therapy for 14 days was accompanied by an increase in muscle protein synthesis by 50% in post-menopausal women [139]. Finally, progesterone, as estrogen, may preserve the regenerative capacity of skeletal muscles by maintaining the number and function of muscle satellite cells [140].

With regard to obesity, in a study by R.M. Whynott et al. [141], it was shown that both BMI and body weight correlated negatively with serum progesterone concentrations. These data were confirmed by the findings from J. Bellver et al. [142] indicating that serum progesterone concentrations decreased with increasing BMI; therefore, underweight or normal weight women had higher progesterone concentrations compared to overweight or obese women. Among others, decreased serum progesterone concentrations in obesity may participate in the negative impact of excess body fat on the overall body composition, particularly skeletal muscle mass.

#### 3.2.3. Androgens

Androgens have anabolic effects on skeletal muscle, which may decline with age. Furthermore, it is well established that obesity is associated with the decreased production and secretion of androgens in men, providing an additional mechanism for the depletion of skeletal muscle mass and deterioration of their function in obesity. On the contrary, high levels of bioavailable serum testosterone may be the factor predisposing to the development of obesity in women [18]. These data are confirmed by the study in 1765 postmenopausal women indicating that high BMI at the moment of the inclusion was associated with elevated serum concentrations of androgen metabolites [143].

Although the precise mechanisms by which androgens affect muscle mass and strength remain unclear, several hypotheses are discussed. First, testosterone may increase muscle protein synthesis via the acceleration of intracellular amino acid reutilization. This, in turn, leads to the enlargement of muscle fibers and muscle hypertrophy [144]. At least in part, such a mechanism of testosterone action may be explained by its ability to upregulate the expression of myogenin and the mammalian target of rapamycin (mTOR) and to inhibit the production of myostatin, a myokine with a negative impact on muscle structure and function [145,146]. Second, the results of in vivo studies also demonstrate that testosterone may stimulate satellite cell division [147]. Third, it is suggested that testosterone is able to increase intracellular concentrations of Ca^2+^ in myoblasts via G-protein cupelled receptor, thus promoting myoblast growth [148]. Finally, androgens were reported to increase IGF-1 expression [149].

Importantly, androgen receptors and glucocorticoid receptors have 79% and 50% homology in the DNA- and ligand-binding domains; therefore, the anti-catabolic activity of testosterone may be explained by interfering with cortisol binding to its receptor or by competition with cortisol–glucocorticoid receptor complexes for DNA-binding sites. Moreover, testosterone is capable of decreasing glucocorticoid receptor expression in muscle tissue [149].

Both estrogen and testosterone have protective effects on mitochondria in muscle tissue, which may be attributed to their direct action on these organelles via binding with either estrogen or androgen receptors localized on them, as well as to indirect action through the regulation of mitochondrial protein expression at the level of nuclear and mitochondrial DNA. Estrogen and testosterone are also able to activate PGC-1α expression in the muscle and to inhibit mitophagy [150,151,152,153].

## 4. Conclusions

In conclusion, considering the high prevalence of obesity worldwide, as well as the increasingly recognized problem of sarcopenic obesity associated with negative health outcomes, clarifying the underlining pathophysiological mechanisms is of great importance. Obesity is characterized by ectopic fat accumulation in skeletal muscles, leading to increased intermuscular fat deposition and the enhancement of intramyocellular lipid droplet generation. Both these processes are accompanied by the deterioration of muscle structure and function, the disturbance of muscle regeneration capacity, and the impairment of energy metabolism. In turn, the above-mentioned negative changes may lead to the shift in myokine profile and, thus, to the formation of the vicious circle facilitating excess fat accumulation, decreasing muscle mass, and aggravating muscle function. Elevated serum GCs levels, as well as decreased serum concentrations of sex steroids associated with obesity, may represent the additional mechanism contributing to the disturbance in muscle metabolic flexibility, oxidative capacity, and decreased muscle energetic efficiency, further promoting the deterioration of muscle function, growth, and regeneration capacity. Taking into account the known sexual dimorphism of skeletal muscles and variations in the response to steroids depending on gender, the mechanisms of skeletal muscle dysfunction in obesity may differ between men and women. The provided data on the complex interaction between skeletal muscle, adipose tissue, and steroids in obesity indicate the possible need for differentiated and individualized approaches to obesity treatment and the correction of the accompanied decrease in muscle mass and function.

## Figures and Tables

**Table 1:** Sex differences in muscle phenotypes.

Parameter	Males	Females	Reference
Muscle fiber type	More glycolytic fibers (fast-twitch) compared to females	More oxidative fibers (low-twitch) compared to males	[118,120]
Satellite cells	Greater number of satellite cells and higher proliferation capacity compared to males	Less number of satellite cells and reduced proliferation capacity compared to females	[121,122]
Susceptibility to atrophy	More susceptible to inflammation-induced atrophy	More susceptible to disuse-induced atrophy	[123,124]
Predominant mechanism of protein degradation	Autophagy	Ubiquitin–proteasome system	[125,126,127]
Age-related shift in myofiber types	Towards type 1 (slow-twitch) dominant composition	No shift occurs	[124]
Top-ranked differentially expressed process during aging	Oxidative phosphorylation	Cell growth	[124]

## Data Availability

Not applicable.
